# The diversity and distribution of pondweeds (*Potamogeton* and *Stuckenia*) in Croatia (South-Eastern Europe) – Insights from national water monitoring

**DOI:** 10.3897/BDJ.14.e208115

**Published:** 2026-09-29

**Authors:** Marija Bučar, Anja Rimac, Vedran Šegota, Nina Vuković, Antun Alegro

**Affiliations:** 1 Division of Botany, Department of Biology, Faculty of Science, University of Zagreb, Zagreb, Croatia Division of Botany, Department of Biology, Faculty of Science, University of Zagreb Zagreb Croatia

**Keywords:** aquatic plants, Balkans, macrophytes, Potamogetonaceae, Water Framework Directive, water vegetation

## Abstract

Pondweeds (*Potamogeton* and *Stuckenia*, family Potamogetonaceae) include ecologically important freshwater macrophytes that serve as bioindicators of waterbody condition. This study presents the first comprehensive and systematic analysis of the diversity and distribution of *Potamogeton* and *Stuckenia* across the entire territory of Croatia, encompassing both the Pannonian and Dinaric Ecoregions. Data were collected through the national surface water monitoring programme conducted between 2016 and 2024, covering 646 running water sites and 42 standing waterbodies. In total, ten *Potamogeton* spp. and a hybrid (P. × cooperi) amongst them and *Stuckenia pectinata* were recorded. Distribution patterns were analysed in relation to ecoregion, catchment area size, hydromorphological alteration, reference vegetation type and co-occurring macrophyte assemblages. *Potamogeton nodosus* proved to be the most frequent and ecologically versatile taxon, recorded across all ecoregions, catchment sizes and reference vegetation types. *Potamogeton* crispus and *S. pectinata* were identified as common species in the Pannonian Ecoregion, while *P. perfoliatus*, *P. berchtoldii* and *P. natans* were mostly recorded in the Continental–Dinaric Subecoregion and natural running waters. *Potamogeton lucens* was most recorded in the Mediterranean–Dinaric Subecoregion. Rare taxa, including *P. coloratus*, *P. gramineus* and P. × cooperi, were documented from only one or two localities. The results are discussed in the context of species ecology and regional hydrology, providing a robust baseline for future macrophyte research and conservation planning in Croatia and the broader south-eastern European region.

## Introduction

Pondweeds, the genus *Potamogeton*, which formerly included the genus *Stuckenia* (family Potamogetonaceae), are freshwater and brackish, submerged and rooted water plants. Although cosmopolitan, the diversity of the whole Potamogetonaceae family is the highest in the Palaearctic (Chambers et al. 2008). Accordingly, there are 22 *Potamogeton* species (Euro+Med 2026) along with numerous hybrids (Kaplan 2010, Kaplan and Fehrer 2013, Aykurt et al. 2017) in Europe. While some pondweeds are characterised by narrower, submerged leaves (e.g. *Potamogeton berchtoldii*, *P. pusillus*, *P. trichoides*, *Stuckenia pectinata*), others develop broad leaves, either solely submerged (e.g. *P. crispus*, *P. lucens*, *P. perfoliatus*) or both submerged and floating (e.g. *P. nodosus*, *P. natans*, *P. gramineus*, *P. coloratus*). Pondweeds, an important part of the aquatic vegetation, function as ecosystem engineers within the aquatic environments: they alter physical and chemical conditions, influence hydrology and sediment dynamics, contribute as primary producers, regulate nutrient cycling and provide structural habitat and shelter for other organisms, thereby shaping overall biodiversity patterns (Bornette and Puijalon 2009, Bornette and Puijalon 2010). Since different taxa have different ecological optima and tolerances (Bučar et al. 2026), they can indicate the health of the environment, i.e. the level of degradation or trophic status of a waterbody (Preston 1995, Tremp and Kohler 1995).

Although Croatia has a long history of botanical research, macrophyte studies gained momentum quite late, in the 1990s. Macrophytes of oligotrophic lakes were quite extensively studied by Blaženčić et al. (1990), Blaženčić and Blaženčić (Blaženčić and Blaženčić 1992, Blaženčić and Blaženčić 1994, Blaženčić and Blaženčić 1995, Blaženčić and Blaženčić 1996) and Alegro et al. (2016). Ranđelović et al. (1993) studied the hydrophilous and hygrophilous vegetation of the Krka River. Research on vascular macrophytes has so far mostly been focused on the distribution of particular aquatic species (Kočić et al. 2014, Rimac et al. 2018, Šegota et al. 2019, Zeko et al. 2020, Rimac et al. 2021), while, with a few exceptions, there is a lack of more comprehensive ecological studies. Kočić et al. (2008) studied the impact of water nutrient concentrations on macrophyte occurrence in canals of eastern Croatia, while the research on the distribution and ecological preferences of invasive *Elodea* species (Bučar et al. 2024) and of *Potamogeton* and *Stuckenia* species (Bučar et al. 2026) encompassed the whole Croatian territory.

This study covers the entire territory of Croatia, taking into account the inherent heterogeneity of its waterbodies and examines the distribution of *Potamogeton* and *Stuckenia* taxa. Additionally, it analyses the distribution patterns of the recorded pondweed taxa in relation to ecoregions and key characteristics of waterbodies (e.g. size, catchment area, degree of degradation, reference vegetation type), while also incorporating data on accompanying species.

## Materials and methods

### Study area

The study area covers the entire territory of Croatia, which comprises two biogeographical regions, the Pannonian and the Dinaric Ecoregion, according to the national methodology for macrophyte surveys (Anonymous 2019), developed in accordance with the requirements of the Water Framework Directive (European Community 2000). The Pannonian Ecoregion (P) corresponds to the continental lowland part of the country, located between the three major rivers – the Sava, Drava and Danube. It is largely composed of alluvial and diluvial plains formed by silicate Quaternary deposits, with isolated mountain massifs reaching elevations of up to 1200 m above sea level. The climate is temperate, featuring warm summers and generally no dry season (Cfb), while the eastern areas are characterised by predominantly hot summers (Cfa) (Beck et al. 2018). The Dinaric Ecoregion includes the Dinarides and the coastal part of Croatia and is further subdivided into the Continental–Dinaric (CD) and Mediterranean–Dinaric (MD) subecoregions. Large parts of the Dinaric Ecoregion are underlain by Mesozoic carbonate rocks, which have developed extensive karst systems. The Continental–Dinaric Subecoregion is characterised by a temperate climate without a dry season and with warm summers (Cfb), along with relatively stable river discharge. In contrast, the Mediterranean–Dinaric Subecoregion has a temperate Mediterranean climate with hot, dry summers (Csa) (Beck et al. 2018), during which some intermittent rivers dry up.

According to the methodology for macrophyte surveys (Anonymous 2019), six reference vegetation types are distinguished in Croatian running waters. They describe the expected vegetation of a running water segment at the ideal level of naturalness and do not incorporate data on the current ecological state, but rather serve as a starting point in evaluating the degradation level. *Rhynchostegium–Fontinalis* (RF) type is a community typical of springs and small to medium-sized fast-flowing mountain and submountain streams on karst substrates. The presence of pondweeds in such a reference vegetation type indicates degradation of the hydromorphology, resulting in slowed water flow and slight eutrophication. The *Nuphar–Myriophyllum* (NM) and *Berula–Nasturtium* (BN) types are typical of small to medium-sized lowland rivers in both the Dinaric and Pannonian Ecoregions. NM is dominated by rooted floating and submerged macrophytes, while BN is dominated by emergent herbaceous plants and aquatic mosses. Different *Potamogeton* species, such as *Potamogeton lucens*, *P. perfoliatus*, *P. nodosus* and *P. natans* are characteristic of NM as well as of the BN of deeper running waters. The *Myriophyllum* (My) type is typical of streams and rivers in both ecoregions, characterised by macrophytes adapted to fast-flowing and fluctuating waters. It is dominated by finely divided, filamentous-leaved species such as *Myriophyllum spicatum* and *Ranunculus* spp., often accompanied by broad-leaved *Potamogeton* species. The broad-leaved pondweed community (*Potamogeton* type – Po) is typical of large and very large lowland rivers in the Pannonian Ecoregion. It is related to the NM type, but is characterised by the dominance of broad-leaved pondweeds (*Potamogeton lucens*, *P. perfoliatus*, *P. nodosus*), corresponding to the magnopotamid growth form. In most large rivers in Croatia, this community has been altered and impoverished due to various hydrotechnical interventions, including bank reinforcement, channel deepening, channel straightening and artificial stabilisation of banks. The *Hydrocharis–Ceratophyllum* (HC) type is dominated by free-floating macrophytes and typically develops in eutrophic to hypertrophic, slow-flowing or stagnant waters of artificial or heavily altered rivers in the eastern Pannonian Ecoregion. In natural rivers, it appears as a degraded stage of the NM type. It is characteristic of turbid waters that have organogenic sediment at the bottom, where poor substrate prevents rooted species, so free-floating plants dominate, often accompanied by *Ceratophyllum demersum* and other shallowly submerged species that can attain high abundance.

### Field sampling

Macrophytes were recorded within the national surface water monitoring scheme, conducted annually from June to September and from 2016 to 2024 to assess the ecological status of waterbodies, as required by the WFD (European Community 2000). Sampling was carried out in 646 running and 42 standing waterbodies following the national methodology for macrophyte sampling which includes running waters with a catchment area > 10 km² (hereafter referred to as watercourses) and lakes with a surface area > 0.5 km², with the exception of certain heavily modified and artificial lakes with a surface area of less than 0.5 km² that are considered significant from the perspective of water management, namely: Sakadaško Lake, Tribalj, Đale, Golubić, Brljan and Gusić Polje (Anonymous 2019). In watercourses, macrophytes were sampled from the bank either by collecting by hand or with a rake on a telescopic pole, depending on water depth. Sampling was carried out along a stretch of at least 50 metres, gradually extended up to 100 metres until no new species appear, which is particularly common in large rivers. This studied watercourse stretch is referred to as a sampling site. In standing waters, macrophytes are sampled along transects, the number and extent of which depend on the size of the sampled waterbody and sampling is performed from a boat using a rake attached to a rope or pole. Macrophytes were identified in the field whenever reliable identification was possible. During the survey, specimens requiring further taxonomic verification, particularly taxonomically challenging narrow-leaved *Potamogeton* species were collected and conserved in 50% ethanol – 2% glycerine solution (ratio 3:1). Collected material was subsequently examined in the laboratory under a stereomicroscope using Casper and Krausch (1981), Preston (1995), Wiegleb and Kaplan (1998), Szoszkiewicz et al. (2010), Van de Weyer et al. (2011a), Van de Weyer et al. (2011b) and Van de Weyer (2018). The nomenclature follows Euro+Med (2026).

### Data collection and analysis

Sampling site data, which include key physical and hydromorphological descriptors used for the national waterbody typology, were obtained from the national macrophyte sampling methodology (Anonymous 2019). Data on watercourses includes the following key environmental characteristics – catchment area size (< 100 km^2^ – small; 100-1000 km^2^ – medium; 1000-10000 km^2^ – large; > 10000km^2^ – very large; x - significant fluctuations in water flow) and the level of hydromorphological alteration (natural, significantly modified or artificial), while data on standing waterbodies include size (small – < 1 km^2^; medium – 1-10 km^2^; large – > 10 km^2^), altitude (lowland – < 200 m a.s.l.; colline – 200-500 m a.s.l.; submontane – > 500 m a.s.l.) and average depth (< 3 m; 3-10 m; 10-15 m; > 15 m). Standing waterbodies are grouped into natural lakes or significantly modified and artificial lakes.

All distribution maps were created using QGIS Desktop 3.4.9 software (Dawson et al. 2026). Descriptive statistical analyses were performed to summarise the distribution patterns of the taxa included in relation to (sub)ecoregions and the key environmental characteristics of the sampled sites. Additionally, co-occurring macrophyte species were analysed and taxa present in more than 30% of sites for each focal species were identified.

As some localities were sampled on multiple occasions, the initial dataset contained pseudoreplicate data. For analyses of environmental parameters across all watercourses in which the studied taxa were recorded, pseudoreplicates were randomly excluded to ensure that each sampling site was represented only once. In contrast, for taxon-specific habitat analyses, the datasets were first filtered to retain all sites at which the respective taxon was recorded, after which pseudoreplicates were excluded. This procedure resulted in unique, species-specific datasets, while preserving the full range of sites associated with each taxon.

## Results

**Figure 1.**
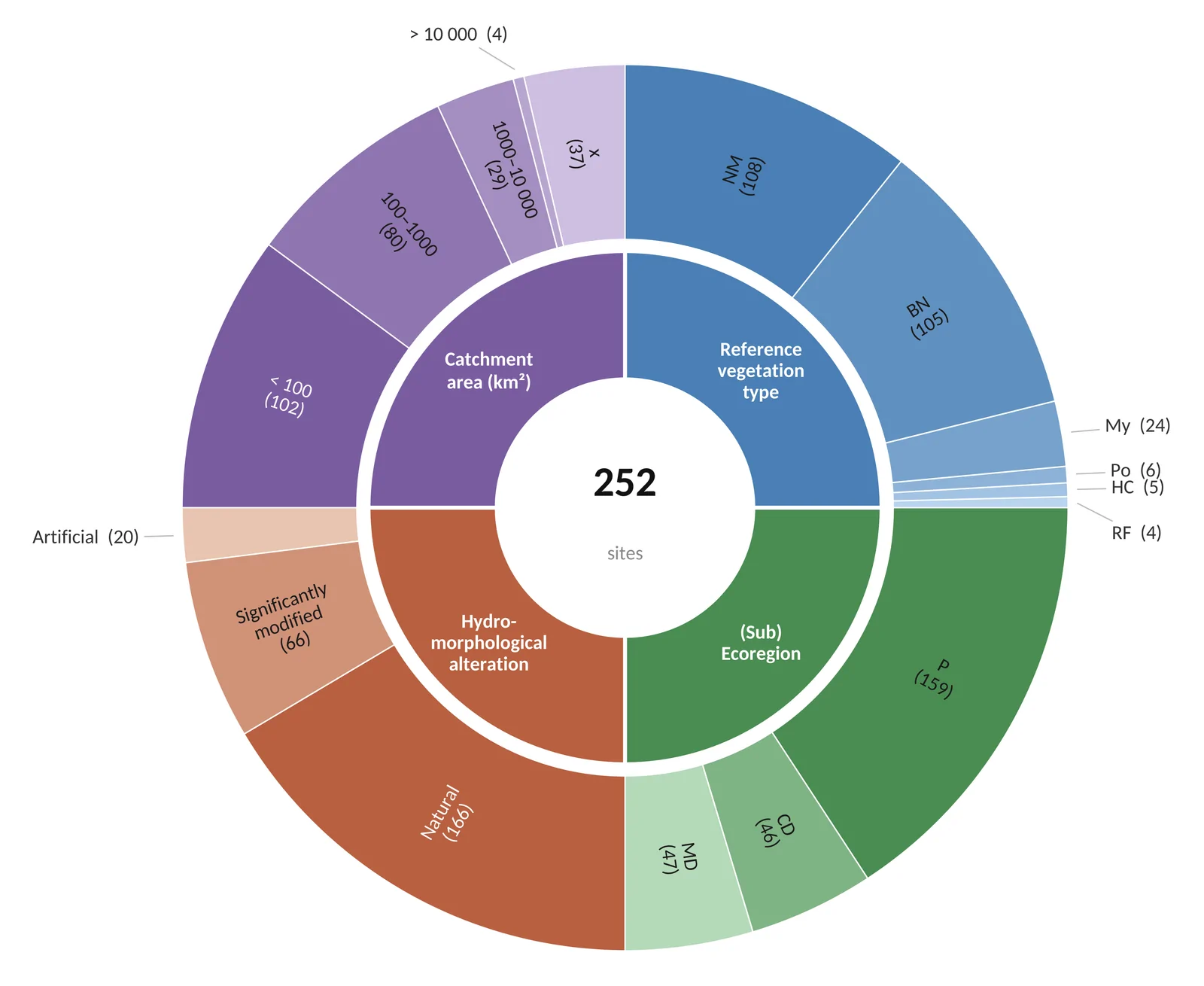
Distribution of watercourse sampling sites with recorded pondweeds across environmental categories (NM – *Nuphar*–*Myriophyllum* type, BN – *Berula*–*Nasturtium* type, My – *Myriophyllum* type, Po – *Potamogeton* type, HC – *Hydrocharis*–*Ceratophyllum* type, RF – *Rhynchostegium*–*Fontinalis* type; P – Pannonian Ecoregion, CD – Continental-Dinaric Subecoregion, MD – Mediterranean-Dinaric Subecoregion; x – significant fluctuations in water flow).

Ten *Potamogeton* species (*Potamogeton nodosus* Poir., *P. crispus* L., *P. perfoliatus* L., *P. berchtoldii* Fieber, *P. lucens* L., *P. natans* L., *P. pusillus* L., *P. trichoides* Cham. & Schltdl., *P. coloratus* Hornem. and *P. gramineus* L.), one *Stuckenia* species (*Stuckenia pectinata* (L.) Börner) and one *Potamogeton* hybrid (Potamogeton × cooperi (Fryer) Fryer) were recorded during the research and analysed with respect to their distribution and environmental variables in 252 watercourses, six natural lakes and 26 significantly modified or artificial lakes. An overview of the sampled watercourses and lakes in which *Potamogeton* and *Stuckenia* taxa were recorded, with key environmental characteristics, is provided in Fig. 1 and Table 1, respectively.

Most watercourses are small or medium-sized (72.2%), natural (65.9%) and situated in the Pannonian Ecoregion (63.1%), with NM and BN reference vegetation type (84.6%). There are very few very large watercourses (1.6%) and those with Po (2.4%), HC (2.0%) and RF (1.6%) reference vegetation type (Fig. 1). Half of the natural lakes are small (50.0%) and deeper than 15 m (50.0%), while more than a half are situated in the lowland (66.7%) of the Mediterranean–Dinaric Subecoregion (66.7%). Most of the significantly modified and artificial lakes are medium-sized (61.5%), 3-10 m deep (61.5%) and situated in the lowlands (69.2%) of the Mediterranean-Dinaric Subecoregion (50.0%) and Pannonian (42.3%) Ecoregion (Table 1).

**Figure 2.**
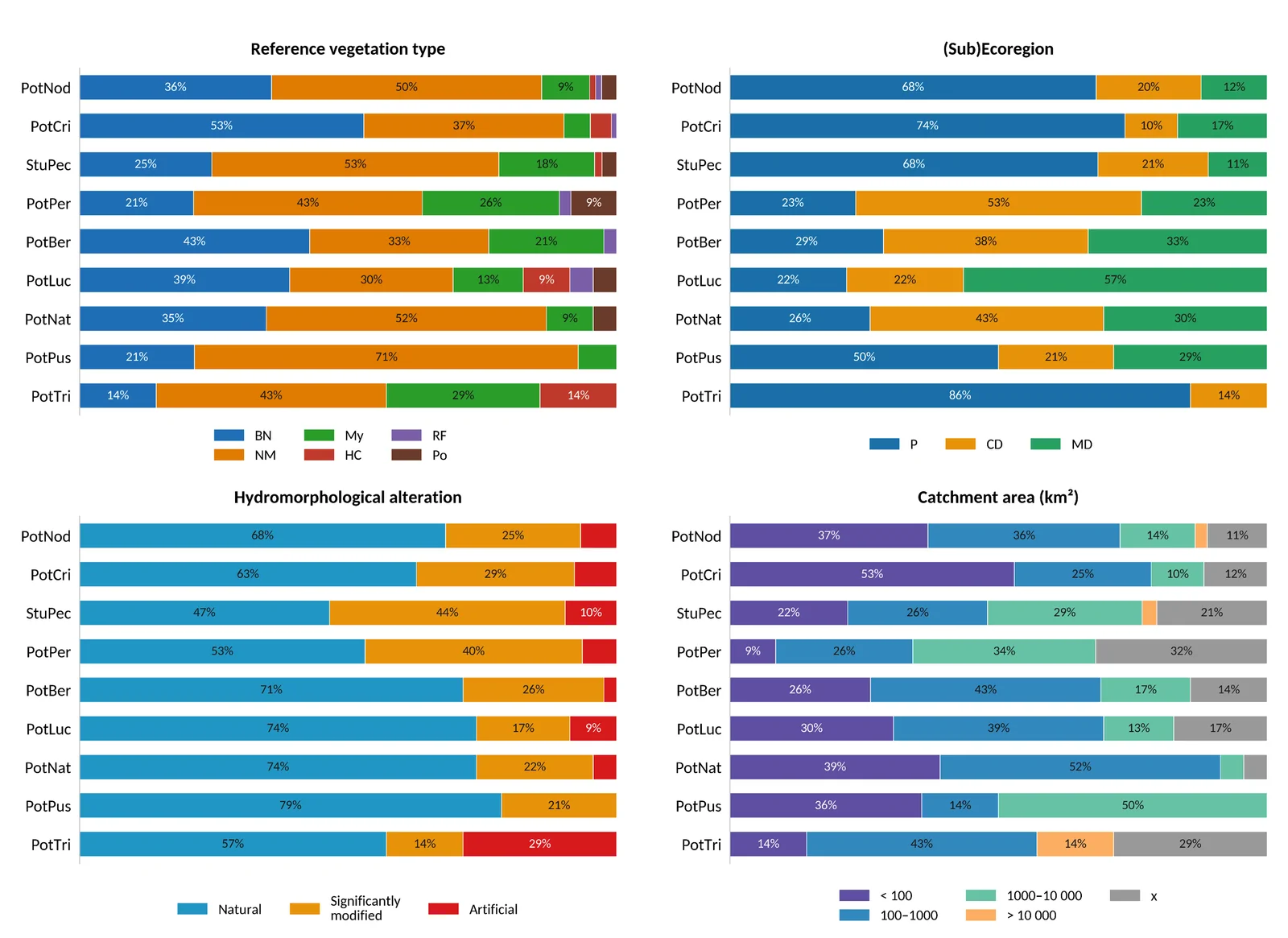
Number and frequency of watercourse sampling sites with pondweed species occurring in more than one locality across different classes of key environmental characteristics (NM – *Nuphar*–*Myriophyllum* type, BN – *Berula*–*Nasturtium* type, My – *Myriophyllum* type, Po – *Potamogeton* type, HC – *Hydrocharis*–*Ceratophyllum* type, RF – *Rhynchostegium*–*Fontinalis* type; P – Pannonian Ecoregion, CD – Continental–Dinaric Subecoregion, MD – Mediterranean–Dinaric Subecoregion; x – significant fluctuations in water flow; PotNod – *Potamogeton nodosus*, PotCri – *Potamogeton crispus*, StuPec – *Stuckenia pectinata*, PotPer – *Potamogeton perfoliatus*, PotBer – *Potamogeton berchtoldii*, PotLuc – *Potamogeton lucens*, PotNat – *Potamogeton natans*, PotPus – *Potamogeton pusillus*, PotTri – *Potamogeton trichoides*).

**Figure 3.**
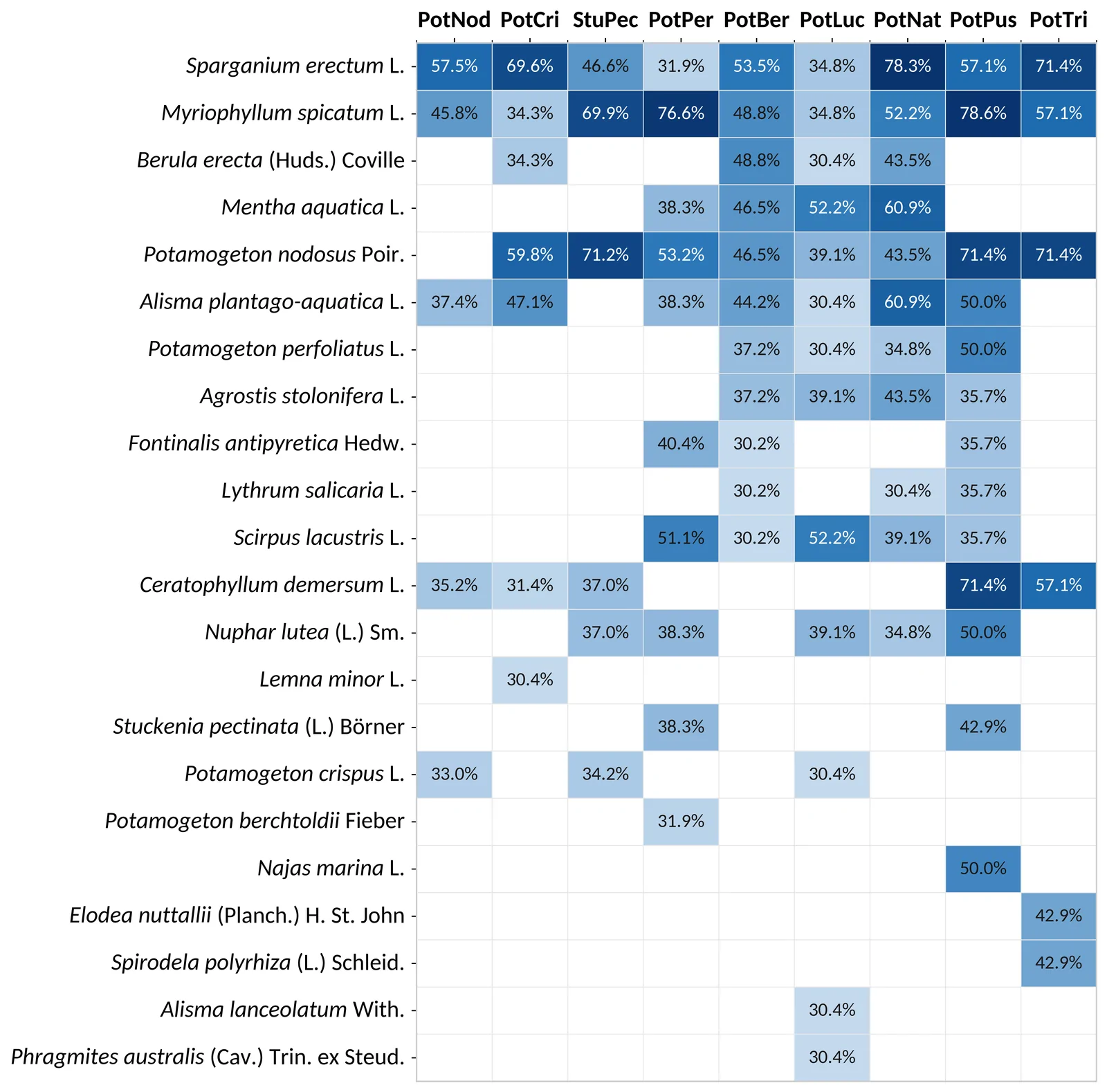
Co-occurrence of aquatic plant species with the investigated taxa. Cell values represent the share of sites where both the studied Potamogetonaceae taxon and the respective co-occurring species were recorded, relative to the total number of sites where the studied Potamogetonaceae taxon was recorded (PotNod – *Potamogeton nodosus*, PotCri – *Potamogeton crispus*, StuPec – *Stuckenia pectinata*, PotPer – *Potamogeton perfoliatus*, PotBer – *Potamogeton berchtoldii*, PotLuc – *Potamogeton lucens*, PotNat – *Potamogeton natans*, PotPus – *Potamogeton pusillus*, PotTri – *Potamogeton trichoides*).

The watercourse sampling sites of the more frequent species provided a comparative insight into their distribution regarding the (sub)ecoregion, the level of hydromorphological alteration, catchment area and the reference vegetation type (Fig. 2, Suppl. material 1). *P. coloratus* and P. × cooperi were found at only one and two watercourse sampling sites, respectively and *P. gramineus* was recorded only in two lakes. Additionally, Fig. 3 provides an overview of species co-occurring in over 30% of watercourses. The full data on the individual running water and standing water localities are given in Suppl. material 2 and Suppl. material 3, respectively. Suppl. material 4 provides a machine-readable format of occurrences of the studied taxa.

### Potamogeton nodosus Poir.

**Figure 4.**
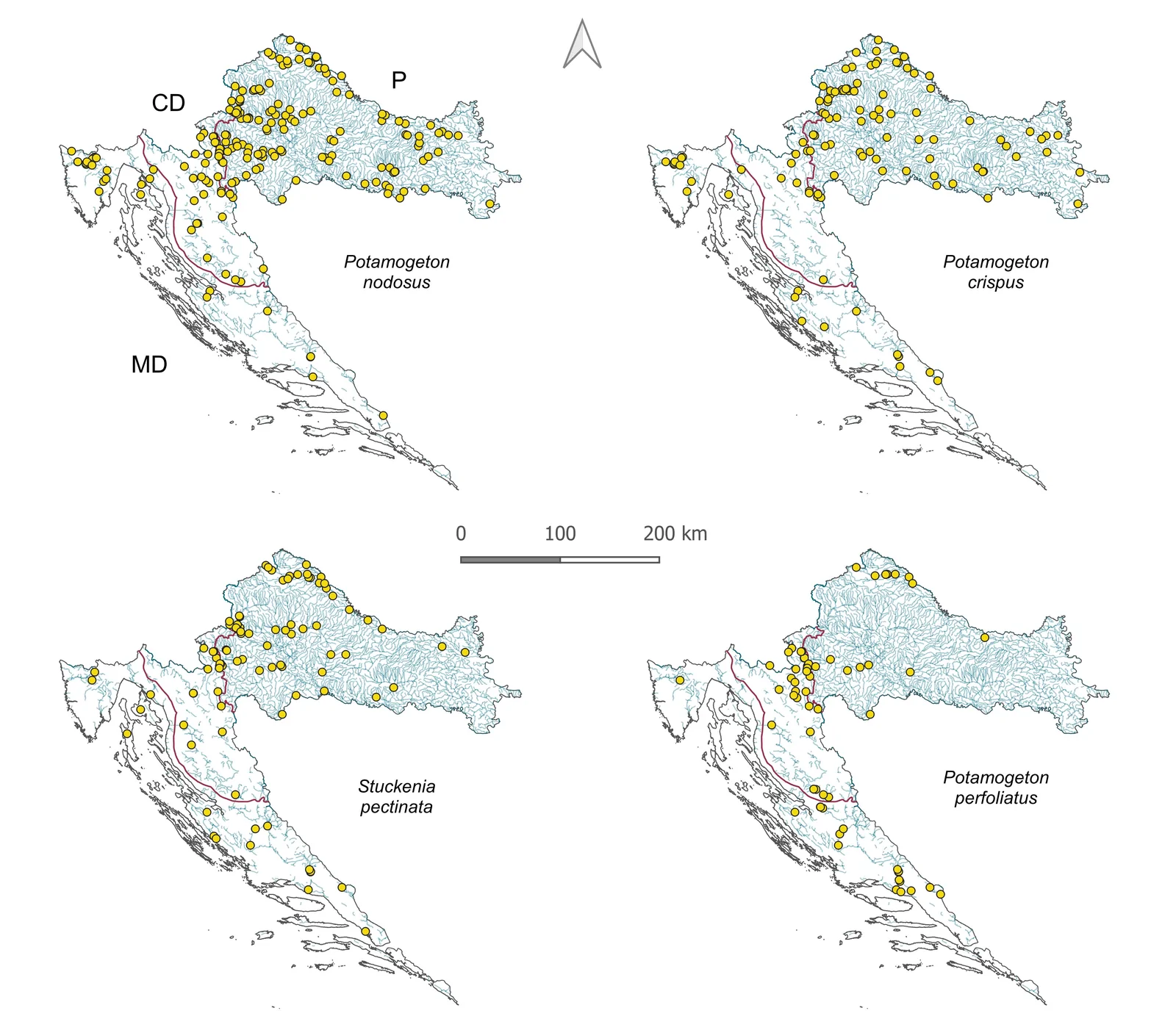
Distribution of *Potamogeton nodosus*, *P. crispus*, *Stuckenia pectinata* and *P. perfoliatus* in Croatia (P - Pannonian Ecoregion, CD – Continental–Dinaric Subecoregion, MD – Mediterranean–Dinaric Subecoregion).

The most frequent *Potamogeton* and *Stuckenia* species in Croatia is *P. nodosus*, occurring in 179 (27.7%) of all investigated watercourse sampling sites (Fig. 2, Suppl. material 1). Although these sites are mostly located in the Pannonian Ecoregion (68.2%), *P. nodosus* is still the most frequent species in the other two Dinaric Subecoregions as well, with 35 (19.6%) records in the Continental–Dinaric Subecoregion and 22 (12.3%) records in the Mediterranean–Dinaric Subecoregion (Fig. 4). Regarding the catchment area, *P. nodosus* is recorded in watercourses of all size; however, it is most frequent in small (< 100 km^2^) and medium-sized (100–1000 km^2^) watercourses, which together account for 72.7% of records. This is consistent with the most frequent reference vegetation types, as *P. nodosus* most frequently occurs in NM and BN (86.1% of records combined, i.e. 50.3% and 35.8%, respectively), typical for small and medium-sized watercourses. Most localities are natural, with no significant hydromorphological alteration (68.2%). The most common accompanying species of *P. nodosus* in watercourses are *Sparganium erectum*, *Myriophyllum spicatum*, *Alisma plantago-aquatica*, *Ceratophyllum demersum* and *Potamogeton crispus* (Fig. 3).

*Potamogeton nodosus* is recorded in one natural (Kozjak within Plitvice Lakes) and 16 significantly modified and artificial lakes, predominantly in the Mediterranean–Dinaric Subecoregion and Pannonian Ecoregion. Most of these standing waterbodies are large, lowland and quite shallow, with depths ranging from 3 to 10 m (Suppl. material 3).

### Potamogeton crispus L.

*P. crispus* is the second most frequent amongst the taxa of interest, recorded in 102 (15.8%) of all investigated watercourse sampling sites (Fig. 2, Suppl. material 1), most of which are located in the Pannonian Ecoregion (73.5%) (Fig. 4). Similarly to *P. nodosus*, it is recorded mostly in small and medium-sized watercourses (78.4% combined); however, it is not recorded in very large rivers. Accordingly, it occurs most frequently in BN and NM reference vegetation types (52.9% and 37.3%, respectively). Most of the recorded localities of this species are natural, with no significant hydromorphological alterations (62.7%). In watercourses, it is most commonly accompanied by *Sparganium erectum*, *Potamogeton nodosus*, *Alisma plantago-aquatica*, *Berula erecta*, *Myriophyllum spicatum*, *Ceratophyllum demersum* and *Lemna minor* (Fig. 3).

*Potamogeton crispus* is recorded in 10 significantly modified and artificial lakes, predominantly in the Mediterranean–Dinaric Subecoregion. Most of the standing waterbodies where it was recorded are large and lowland, while their average depth is less than 10 m (Suppl. material 3).

### Stuckenia pectinata (L.) Börner

*S. pectinata* is the third most frequent pondweed, recorded in 73 (11.3%) of all investigated watercourse sampling sites (Fig. 2, Suppl. material 1). It is mostly recorded in the Pannonian Ecoregion (68.5%) (Fig. 4) and most frequent in large (28.8%) watercourses; however, it is also quite common in medium-sized and small watercourses (21.9% each), as well as in watercourses with significant fluctuations in water flow (20.5%). Accordingly, *S. pectinata* most frequently occurs in NM reference vegetation type (53.4%), typical for medium and large watercourses. This species, alongside *P. nodosus*, occurs in watercourses of all catchment area size classes. With regard to hydromorphological alteration, *S. pectinata* is the only pondweed with less than half of its records from natural watercourses (46.6%), while showing an almost even distribution in significantly modified watercourses (43.8%). The most common accompanying species of *S. pectinata* in watercourses are *Potamogeton nodosus*, *Myriophyllum spicatum*, *Sparganium erectum*, *Ceratophyllum demersum*, *Nuphar lutea* and *Potamogeton crispus* (Fig. 3).

*S. pectinata* is recorded in five natural and 14 significantly modified and artificial lakes, predominantly in the Mediterranean–Dinaric Subecoregion. Most of the standing waterbodies where it was recorded are large and lowland, while their average depth is less than 10 m (Suppl. material 3).

### Potamogeton perfoliatus L.

*P. perfoliatus* is recorded in 47 (7.3%) of all investigated watercourse sampling sites (Fig. 2, Suppl. material 1), mostly in the Continental–Dinaric Subecoregion (53.2%) and equally in the Pannonian Ecoregion and the Mediterranean–Dinaric Subecoregion (23.4% each) (Fig. 4). It is recorded most frequently in large watercourses (34%), in watercourses with significant fluctuations in water flow (31.9%) and in medium-sized watercourses (25.5%). It is rarely recorded in small and is absent from very large watercourses. From the point of view of vegetation, *P. perfoliatus* inhabits NM sites most frequently (42.6%), followed by My (25.5%) and BN (21.3%). It occurs most frequently in natural watercourses (53.2%), being quite common, however, in significantly modified watercourses (40.4%), similarly to *S. pectinata*. The most common accompanying species of *P. perfoliatus* in watercourses are *Myriophyllum spicatum*, *Potamogeton nodosus*, *Scirpus lacustris*, *Fontinalis antipyretica*, *Stuckenia pectinata*, *Alisma plantago-aquatica*, *Mentha aquatica*, *Nuphar lutea*, *Potamogeton berchtoldii* and *Sparganium erectum* (Fig. 3).

*P. perfoliatus* is recorded in two natural and 14 significantly modified and artificial lakes, predominantly in the Mediterranean–Dinaric Subecoregion. It is recorded mostly in lowland lakes with an average depth of 3-10 m and is equally distributed between small and large lakes (Suppl. material 3).

### Potamogeton berchtoldii Fieber

**Figure 5.**
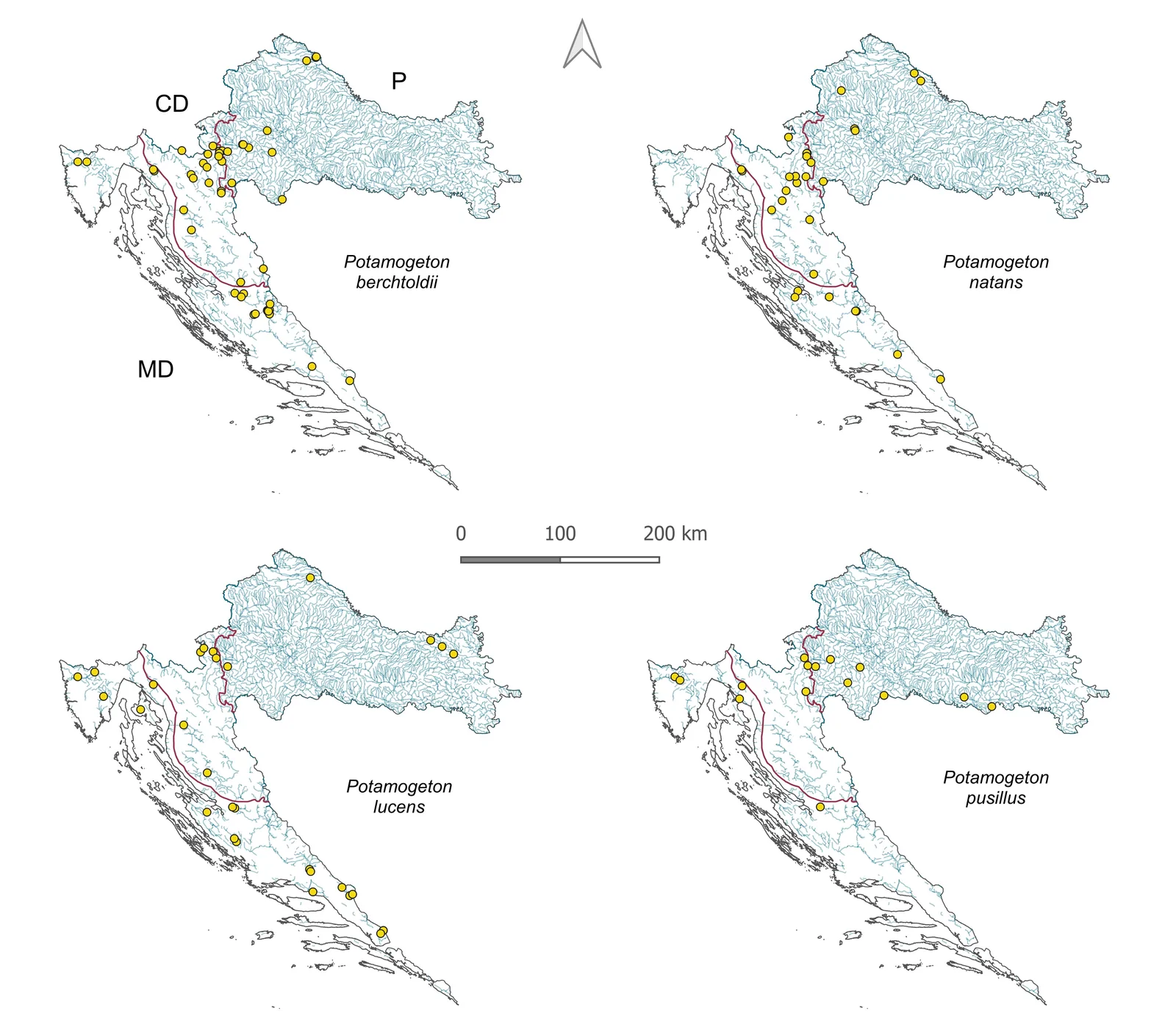
Distribution of *Potamogeton berchtoldii*, *P. natans*, *P. lucens* and *P. pusillus* in Croatia. (P – Pannonian Ecoregion, CD – Continental–Dinaric Subecoregion, MD – Mediterranean–Dinaric Subecoregion).

*P. berchtoldii* is recorded in 42 (6.5%) of all investigated watercourse sampling sites (Fig. 2, Suppl. material 1), mostly in the Continental–Dinaric Subecoregion (38.1%) and almost equally in the Mediterranean–Dinaric Subecoregion (33.3%) and the Pannonian Ecoregion (28.6%) (Fig. 5). It is mostly recorded in medium-sized and small watercourses (42.9% and 26.2%, respectively), most frequently within reference vegetation types BN (42.9%), NM (33.3%) and My (21.4%). *P. berchtoldii* is amongst four species which are significantly more frequent (> 70%) in natural than in significantly modified watercourses, together with *P. lucens*, *P. natans* and *P. pusillus* The most common accompanying species in watercourses are *Sparganium erectum*, *Myriophyllum spicatum*, *Berula erecta*, *Mentha aquatica*, *Potamogeton nodosus*, *Alisma plantago-aquatica*, *Potamogeton perfoliatus*, *Agrostis stolonifera*, *Fontinalis antipyretica*, *Lythrum salicaria* and *Scirpus lacustris* (Fig. 3).

*P. berchtoldii* is recorded in five significantly modified and artificial lakes, of which three are located in the Mediterranean–Dinaric and two in the Continental–Dinaric Subecoregion. These lakes are mostly small and colline, with an average depth of less than 10 m (Suppl. material 3).

### Potamogeton lucens L.

*P. lucens* is recorded in 23 (3.7%) of all investigated watercourse sampling sites (Fig. 2, Suppl. material 1), mostly situated in the Mediterranean–Dinaric Subecoregion (56.5%) and equally distributed in the Pannonian Ecoregion and Continental–Dinaric Subecoregion (five records, i.e. 21.7% each) making it the species which is most frequent in the Mediterranean–Dinaric Subecoregion (Fig. 5). It is recorded most frequently in medium and small watercourses (nine and seven records, respectively), followed by watercourses with significant fluctuations in water flow (four records) and large watercourses (three records). Similarly to *P. nodosus*, *P. lucens* occurs in all reference vegetation types, prevalently in BN (nine records) and NM (seven records). Like *P. berchtoldii, P. lucens* is also mostly observed in natural watercourses (17 records). The most common accompanying species in watercourses are *Mentha aquatica*, *Scirpus lacustris*, *Potamogeton nodosus*, *Agrostis stolonifera*, *Nuphar lutea*, *Myriophyllum spicatum*, *Sparganium erectum*, *Potamogeton crispus*, *Potamogeton perfoliatus*, *Alisma lanceolatum*, *Alisma plantago-aquatica*, *Berula erecta* and *Phragmites australis* (Fig. 3).

*Potamogeton lucens* is recorded in six significantly modified and artificial lakes, most of which are located in the Mediterranean–Dinaric Subecoregion. The lakes are predominantly small and colline, 3-10 m deep (Suppl. material 3).

### Potamogeton natans L.

*P. natans* is recorded in 23 (3.7%) of all investigated watercourse sampling sites (Fig. 2, Suppl. material 1), mostly in the Continental–Dinaric Subecoregion (10 sites, 43.5%) and almost equally in the Mediterranean–Dinaric Subecoregion (seven sites, 30.4%) and the Pannonian Ecoregion (six sites, 26.1%), similarly to *P. berchtoldii*, but with fewer records (Fig. 5). *P. natans* most frequently occurs in medium sized-watercourses (12 records), followed by small watercourses (nine records). Accordingly, the most frequent reference vegetation type in which it is recorded is NM (12 records), followed by BN (eight records). Like *P. berchtoldii* and *P. lucens*, *P. natans* also prefers natural watercourses (17 records). The most common accompanying species of *P. natans* in watercourses are *Sparganium erectum*, *Alisma plantago-aquatica*, *Mentha aquatica*, *Myriophyllum spicatum*, *Potamogeton nodosus*, *Agrostis stolonifera*, *Berula erecta*, *Scirpus lacustris*, *Potamogeton perfoliatus*, *Nuphar lutea* and *Lythrum salicaria* (Fig. 3).

*P. natans* is recorded in one natural (Prošćansko jezero, Plitvice Lakes) and four significantly modified and artificial lakes, three of which are located in the Mediterranean–Dinaric Subecoregion and two in the Continental–Dinaric Subecoregion. The lakes are small and predominantly submontane, with an average depth of less than 10 m (Suppl. material 3).

### Potamogeton pusillus L.

*P. pusillus* is recorded in 14 (2.2%) of all investigated watercourse sampling sites (Fig. 2, Suppl. material 1), seven (50.0%) of which are in the Pannonian Ecoregion, four in the Mediterranean–Dinaric Subecoregion (28.6%) and three in the Continental–Dinaric Subecoregion (21.4%) (Fig. 5). It occurs in seven large, five small and two medium-sized watercourses. Most of its records are from sites with the NM vegetation type (10 records) and is completely absent from HC, RF and Po sites. Like the previous three species, *P. pusillus* is recorded in natural watercourses with more than 70% of occurrences (11 records) and is absent from artificial watercourses. The most common accompanying species in watercourses are *Myriophyllum spicatum*, *Potamogeton nodosus*, *Ceratophyllum demersum*, *Sparganium erectum*, *Potamogeton perfoliatus*, *Alisma plantago-aquatica*, *Najas marina*, *Nuphar lutea*, *Potamogeton pectinatus*, *Agrostis stolonifera*, *Fontinalis antipyretica*, *Lythrum salicaria* and *Scirpus lacustris* (Fig. 3).

*P. pusillus* is recorded only in one significantly modified and artificial lake (Butoniga), located in the Mediterranean–Dinaric Subecoregion. Butoniga is a large, lowland lake, 3-10 m deep (Suppl. material 3).

### Potamogeton trichoides Cham. & Schltdl.

**Figure 6.**
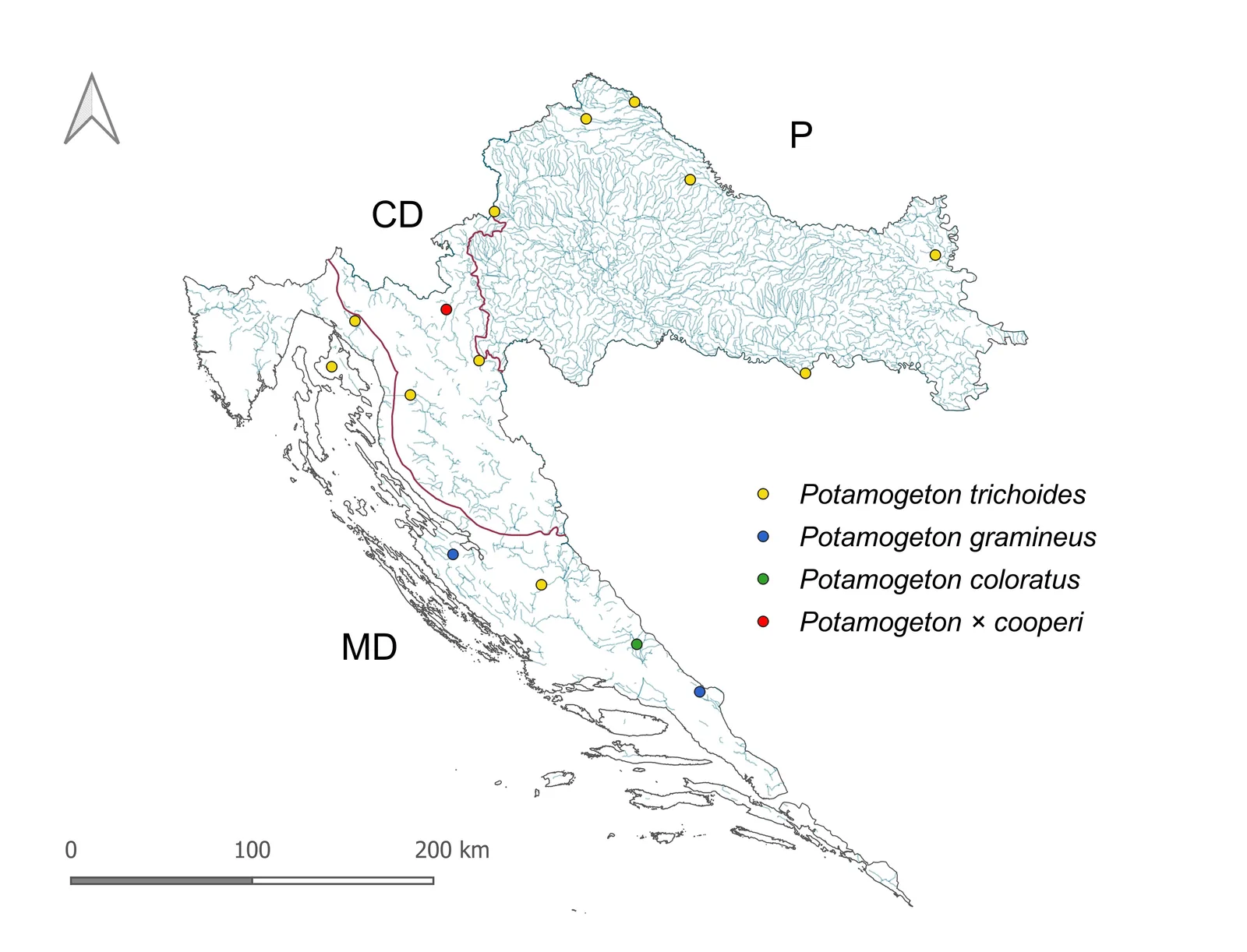
Distribution of *Potamogeton trichoides*, *P. gramineus*, *P. coloratus* and P. × cooperi in Croatia (P – Pannonian Ecoregion, CD – Continental–Dinaric Subecoregion, MD – Mediterranean–Dinaric Subecoregion).

*P. trichoides* is recorded in only seven (1.0%) of the investigated watercourse sampling sites (Fig. 2, Suppl. material 1), with six sites located in the Pannonian Ecoregion and one in the Continental–Dinaric Subecoregion (Fig. 6). Three records are from medium-sized watercourses, two from watercourses with significant fluctuations in water flow and one each from small and very large watercourses. In terms of vegetation, most sites belong to NM (three records) and My (two records) reference vegetation type. Regarding hydromorphological alteration, four sites are natural, two artificial and one is a significantly modified watercourse. The most common accompanying species in watercourses are *Potamogeton nodosus*, *Sparganium erectum*, *Ceratophyllum demersum*, *Myriophyllum spicatum*, *Elodea nuttallii* and *Spirodela polyrhiza*, each recorded in three or more of *P. trichoides* localities (Fig. 3).

*P. trichoides* is recorded in four significantly modified and artificial lakes, most of which are small and located in the Mediterranean–Dinaric Subecoregion. Two lakes have a mean depth below 3 m, while the remaining two have mean depths between 3 and 10 m. Regarding altitude, two lakes are lowland, one is colline and one submontane (Suppl. material 3).

### Potamogeton gramineus L.

*P. gramineus* is only recorded in two significantly modified and artificial lakes, Vlaćine and Prološko Blato. Both lakes have average depths between 3 and 10 m and are situated in the Mediterranean–Dinaric Subecoregion (Fig. 6). Vlaćine is a small, lowland lake, while Prološko Blato is a large, colline lake. Other pondweeds which grow in both lakes are *Potamogeton crispus*, *P. perfoliatus*, *P. lucens* and *S. pectinata* (Suppl. material 3).

### Potamogeton coloratus Hornem.

*P. coloratus* is recorded only in Milanovići Canal. This is an artificial, Mediterranean–Dinaric watercourse with BN reference vegetation type which discharges into the river Cetina (Fig. 6, Suppl. material 2). It has significant seasonal fluctuations in water flow and occasionally dries out during the warmest part of the year. It is accompanied by *Callitriche platycarpa*, *Lythrum salicaria*, *Scirpus lacustris*, *Sparganium erectum*, *Typha latifolia*, *Veronica anagallis-aquatica*, *Alisma plantago-aquatica* and *Nasturtium officinale*.

### Potamogeton × cooperi (Fryer) Fryer

P. × cooperi is a hybrid of *P. perfoliatus* and *P. crispus*, recorded only in two close localities on the River Dobra (Dobra 2 and Dobra 3), which is a significantly modified, Continental–Dinaric watercourse with significant fluctuations in water flow and My reference vegetation type (Fig. 6, Suppl. material 2). Both localities are situated downstream from a hydropower plant that causes periodical changes in the water flow; however, the Dobra never dries out. It is accompanied by the parental species *P. perfoliatus* and *P. crispus* and also *Fontinalis antipyretica*, *Callitriche platycarpa*, *Elodea canadensis*, *Myosotis scorpioides*, *Myriophyllum spicatum*, *Potamogeton berchtoldii*, *Agrostis stolonifera*, *Cinclidotus fontinaloides*, *Cinclidotus riparius*, *Nasturtium officinale*, *Rhynchostegium riparioides* etc.

## Discussion

When discussing the overall distribution of *Potamogeton* and *Stuckenia* in Croatia and also regarding the ecoregions, the general distribution of waterbodies in Croatia, especially watercourses, should be emphasised. The Pannonian Ecoregion is geographically most favourable for a large number of watercourses due to its impermeable silicate substrate, low-relief topography and climate with even precipitation year-round, without a drought period. The Continental–Dinaric Subecoregion also supports perennial rivers due to its climate with abundant precipitation; however, the mountainous, carbonate terrain favours smaller rivers with stronger water flow. Additionally, due to the rough terrain and lower demographic density, these rivers are often comparably less disturbed and polluted. The climate of the Mediterranean–Dinaric Subecoregion is warm, with summer drought periods, which contribute to the intermittent nature of some smaller rivers; however, larger karst rivers also occur and, together with numerous standing waterbodies, support aquatic vegetation throughout the year. In summary, due to the abundance of waterbodies in the Pannonian Ecoregion, the majority of the sampled watercourses are small and medium-sized rivers with *Berula–Nasturtium* (BN) and *Nuphar–Myriophyllum* (NM) reference vegetation type. Finally, very large rivers, such as the Danube, Drava and Sava, are excluded from the macrophyte monitoring due to severe anthropogenic degradation associated with their high economic value and intensive use and, correspondingly, lack of macrophyte communities. This largely explains the small number of the *Potamogeton* vegetation type within the study, although this type is naturally characterised by large-leaved *Potamogeton* species and is typical for large and very large rivers.

*P. nodosus* is the most frequently occurring pondweed in Croatia, as well as within each individual (sub)ecoregion. This can be attributed to its wide ecological tolerance. It is present in watercourses of all reference vegetation types and all categories of catchment areas. Although *P. nodosus* co-occurs with a relatively high number of species, only a few exhibit high co-occurrence frequencies with *P. nodosus* (Fig. 3), which is consistent with its low habitat specificity. On the other hand, *P. nodosus* is frequently found accompanying other pondweed taxa. It is also the only pondweed which is amongst the most frequent accompanying species of all other studied taxa. A previous ecological study on *Potamogeton* species in Croatia, which incorporated physical and chemical water parameters, showed that *P. nodosus* is amongst the most ecologically tolerant pondweeds with respect to total phosphorus and nitrogen, electrical conductivity, pH and temperature (Bučar et al. 2026). Its ecological versatility is confirmed by multiple studies (Maissour and Benamar 2019, Bayındır and İkinci 2020). When compared to *P. natans*, the most frequently recorded *Potamogeton* species in Europe (Global Biodiversity Information Facility 2026), *P. nodosus* is less frequent in Central Europe and absent from most parts of Northern Europe (Schou et al. 2023, Euro+Med 2026), indicating its thermophilic nature. Globally, however, *P. nodosus* is a true cosmopolite, whereas *P. natans* is mostly distributed in the Holarctic (POWO 2026).

The second most frequent pondweed is *P. crispus*, which is recorded in about one third of *P. nodosus* localities. This species is an indicator of eutrophication and disturbance, which thrives in highly conductive water, even under conditions of low dissolved oxygen (Wiegleb et al. 1991, O’Hare et al. 2006, Zelnik et al. 2021, Bučar et al. 2026). It is most often recorded in small and medium-sized rivers of the Pannonian Ecoregion, within the predominant BN and NM vegetation type. However, its presence in such rivers indicates ecological degradation, i.e. eutrophication. Additionally, *P. crispus* is also a strong competitor, which develops turions in autumn and displays rapid growth early in the vegetation season, while the water temperature is still relatively low and other submerged plants are still dormant (Kunii 1982, Tobiessen and Snow 1984). In the latter study, the stems of *P. crispus* started proliferating even in the low light conditions, indicating a low-light compensation point, which can be advantageous in turbid watercourses.

*Stuckenia pectinata* has a similar distribution to *P. crispus* in watercourses in Croatia. Generally, *S. pectinata* is a cosmopolitan species, most commonly growing in the temperate regions (POWO 2026, Global Biodiversity Information Facility 2026). It has an extremely wide ecological amplitude, occurring in various freshwater and brackish, running and standing aquatic habitats (Van Wijk 1988). It is also an indicator of eutrophication and ecological degradation (Poikane et al. 2018). In Croatia, *S. pectinata* thrives in turbid, nutrient-rich watercourses, even under conditions of low dissolved oxygen (Bučar et al. 2026). However, this species is also recorded in most of the studied lakes, precisely five out of six natural lakes, which are oligotrophic and mesotrophic, testifying to the versatile nature and competitiveness of *S. pectinata*, but also to the ecological degradation in terms of eutrophication (Alegro et al. 2016).

Even though the three previously discussed pondweeds have similar distributions and ecological tolerances and are often recorded together, *P. nodosus* is evidently more frequent in the study. The reasons behind its competitiveness could be morphological and, subsequently, physiological. While *P. crispus* and *S. pectinata* develop only submerged leaves (ovate and narrowly-lanceolate, respectively), *P. nodosus* has broad leaves, both submerged and floating. Heterophyllous plants can efficiently take up nutrients from the water column, while also shading out the competing submerged plants, accessing the atmospheric CO₂, increasing direct interception of photosynthetically active radiation and facilitating gas exchange at the ventral lamina of their floating leaves (Wells and Pigliucci 2000, Shabnam and Pardha-Saradhi 2016, Li et al. 2019).

Three pondweed taxa, *P. perfoliatus*, *P. berchtoldii* and *P. natans* are mostly recorded in the Continental–Dinaric Subecoregion and frequently co-occur. The most frequent amongst these, *P. perfoliatus*, is the only pondweed with more than half of its records from that region. It is more frequent in large watercourses and those of medium size where the reference vegetation types are NM and My. Additionally, it is almost equally present in natural and significantly modified watercourses. The latter distinguishes it from *P. berchtoldii* and *P. natans*, which are both mostly recorded in natural, small to medium sized watercourses with BN and NM reference vegetation types. Unlike *P. berchtoldii* and *P. natans*, *P. perfoliatus* is present in as many as 16 standing waterbodies from all three ecoregions. It is noteworthy that *P. perfoliatus* is recorded in both natural and significantly modified waterbodies, as it has the narrowest ecological tolerance amongst pondweeds in Croatia (Bučar et al. 2026). According to Bučar et al. (2026), all three species exhibit similar ecological tolerances with respect to dissolved oxygen (DO), nutrients, electrical conductivity and pH. They favour high DO, low to moderate nutrient concentrations (total phosphorus and total nitrogen) and moderate electrical conductivity (optimum at 350 μS/cm). However, *P. natans* stands out from the group by not occurring at pH values above 7.8, whereas the other two species clearly favour alkaline waters. There is little difference in the distribution of *P. berchtoldii* and *P. natans*, although the former is almost twice as frequently recorded overall, occurring more frequently in BN than in NM sites.

*P. lucens* is predominantly found in the Mediterranean–Dinaric Subecoregion, within which the majority of its records in this study have been documented. It most commonly inhabits small to medium-sized natural watercourses, particularly those with BN and NM vegetation types; however, it is present across all reference vegetation types, in a pattern similar to *P. nodosus*. The widespread occurrence of permeable carbonate bedrock in the Mediterranean–Dinaric Subecoregion creates chemically favourable conditions for calciphilous species, supporting the distribution and ecological success of *P. lucens*. The species is commonly recorded in waterbodies with high electrical conductivity (EC) and is, therefore, linked to calcareous, mineral-rich waters in different European regions (Preston 1995, Merdalski et al. 2019, Germ et al. 2021). Its ecological success in such habitats is largely due to its physiological capacity to utilie bicarbonate (HCO₃⁻) as an alternative inorganic carbon source even under CO₂-limited conditions. This adaptation provides a competitive advantage over species more strictly dependent on dissolved CO₂, particularly in alkaline, carbonate-rich environments and in dense vegetation stands where CO₂ availability is reduced (Prins et al. 1980, Elzenga and Prins 1988, Miedema and Prins 1992, Kahara and Vermaat 2003).

Due to the low number of records, no robust conclusions can be drawn regarding the distribution of the less frequent taxa, such as *P. pusillus*, *P. trichoides*, *P. gramineus*, *P. coloratus* and P. × *cooperi*. *P. pusillus* and *P. trichoides* are narrow-leaved species, morphologically similar to *P. berchtoldii*, which was once encompassed in the *P. pusillus* aggregate (Kaplan and Štěpánek 2003). Therefore, all of the narrow-leaved pondweed specimens were collected and carefully identified under a stereomicroscope using the literature previously listed in Materials and Methods. *P. berchtoldii* is differentiated from the other two by the two prominent nodal glands, whereas *P. trichoides* is unique by virtue of its very acute leaves with strong, elevated mid-rib (Casper and Krausch 1981, Van de Weyer 2018). Even though all three species are found across a range of habitats and largely share ecological requirements, *P. pusillus* and *P. trichoides* are more frequently found in eutrophic water, with *P. pusillus* additionally often inhabiting slow-flowing watercourses (Preston 1995). This is in accordance with our finding that the latter species are more frequent in the nutrient-rich watercourses of the Pannonian Ecoregion, whereas *P. berchtoldii* favours the Continental–Dinaric Subecoregion. Additionally, half of the records of *P. pusillus* in our study were from large watercourses, which are usually characterised by slower water flow.

*P. gramineus* has been recorded in only two lakes in the Mediterranean–Dinaric Subecoregion. This circumpolar species, present even in the northernmost parts of Europe, typically grows in nutrient-poor to moderately eutrophic lakes with weakly acidic to alkaline water; however, it can also occur in running water (Pip 1987, Wiegleb and Kaplan 1998, Johnsen et al. 2021, Schou et al. 2023). It is characterised by heterophylly and is considered to be extremely polymorphic, to the extent of developing terrestrial forms, which enhances its tolerance to water level fluctuations and periods of drought (Spencer and Ksander 1992, Wiegleb and Kaplan 1998, Bayındır and İkinci 2020). This is particularly relevant to our study, since one of the lakes where it was recorded, Prološko Blato, undergoes periodic drawdowns, with the terrestrial stands of *P. gramineus* growing in shallower areas that may dry out almost completely, while the second lake, Vlaćine, is periodically drained. Other sources indicating the distribution of this species in Croatia include one herbarium specimen from Turopolje area from the late 19^th^ century from the Herbarium Croaticum (ZA) (ZA 12118) (Nikolić et al. 2026) and a few literature records placing it in Kopački rit Nature Park (Panjković 1989, Rožac et al. 2018), Plitvice Lakes National Park (Krga 1992), the island of Mljet (Boršić et al. 2009) and the Pantan impoundment close to the City of Trogir (Vladović et al. 2015).

*P. coloratus* has only been recorded in one locality, in the Mediterranean–Dinaric Subecoregion. Similarly to *P. lucens*, it also grows in calcareous, alkaline water; however, unlike the former, it is unable to use bicarbonate ions from the water, which restricts it to a CO_2_ rich environment (Schou et al. 2023), being subsequently much rarer. According to multiple studies (Carbiener et al. 1990, Amoros et al. 2000, Trémolières 2004, Kaplan et al. 2014), *P. coloratus* inhabits oligotrophic and undisturbed or only slightly disturbed waters. Due to its presence in shallow and slow-flowing waterbodies (Lukács et al. 2020), the distribution of this species is not well captured by the WFD monitoring scheme in Croatia, which targets watercourses with a catchment area > 10 km² and standing waters with a surface area > 0.5 km² with previously noted exceptions. Consequently, smaller ponds, ditches and pools in the Mediterranean–Dinaric Subecoregion remain under-represented in the monitoring network and their inclusion would perhaps improve the understanding of the species’ distribution in Croatia. Ultimately, the current lack of literature and observation on this species indicates its rarity in the aquatic flora of Croatia. Other sources indicating the distribution of this species in Croatia include two herbarium specimens from the island of Rab in 1910 (ZA 12079) and from the River Gacka in 1949 (ZA 12132) (Nikolić et al. 2026) and two literature records from the island of Krk (Trinajstić 1965) and from Kopački rit Nature Park (Rožac et al. 2018).

Potamogeton × cooperi is the first molecularly confirmed pondweed hybrid from Croatia (Zalewska-Gałosz 2022). Morphologically, however, this taxon was first recognised in 2016 in the same locality from the previous study, after which it was confirmed in the following years (2018, 2022 and 2025), together with the parental species, *P. crispus* and *P. perfoliatus*. This consistency indicates stable populations and favourable environmental conditions.

Although this study is grounded in long-term monitoring of aquatic vegetation across a wide range of lentic and lotic habitats, additional sources also contribute to our understanding of the distribution of *Potamogeton* and *Stuckenia* taxa in Croatia. A comprehensive analysis of herbarium collections of aquatic plants documented more than 270 specimens of pondweeds and identified *P. nodosus* and *P. crispus* as the most frequently collected taxa, reflecting their broad distribution in Croatian aquatic systems (Zeko et al. 2020). In this study, three rare and Data Deficient pondweed species (Nikolić and Topić 2005) were recorded: *P. alpinus*, *P. compressus* and *P. polygonifolius*, represented by single herbarium sheets that constitute their only known records in Croatia (Zeko et al. 2020). As these three specimens date back to the late 19^th^ century, their identifications were subsequently verified by the experienced taxonomist Z. Kaplan (Nikolić et al. 2026). *P. alpinus* is amongst the most widely distributed aquatic macrophytes in Central and Northern Europe, although its occurrence becomes increasingly fragmented towards southern parts of the continent (Schou et al. 2023). The species has not been recorded from the entire lowland Banat Region along the Serbia–Romania border (Neacşu et al. 2024), while in Poland, it is considered rare in the northern lowlands (Robionek et al. 2015). In Slovenia, populations in Lake Bohinj have been reported to be declining (Gaberščik and Mazej 1995). Ecologically, the species is typically associated with oligotrophic, stagnant or slow-flowing waters that are neutral to mildly acidic (Preston 1995, Søndergaard et al. 2010, Schou et al. 2023). Similarly, *P. compressus* is a circumboreal species with a scattered distribution across Central and Northern Europe, where it primarily inhabits larger lakes and watercourses (Schou et al. 2023). However, it is absent from the Mediterranean Region and Hungary and remains Data Deficient in Slovenia (Birkinshaw et al. 2013). Herbarium records of both species originate from the same general area, namely the Lonja River and the adjacent Lonjsko Polje wetland complex (Nikolić et al. 2026). Considering the ecological requirements of these taxa and their affinity for cooler climatic conditions, their continued persistence in Croatia appears unlikely, particularly in light of ongoing climatic warming and the resulting decline in habitat suitability for boreal species. *P. polygonifolius* is a sub-Atlantic species typically occurring in oligotrophic, neutral to strongly acidic waters of bogs, small lakes, ditches and streams (Schou et al. 2023). As the herbarium specimen lacks locality data, any assessment of its present-day occurrence in Croatia remains highly uncertain (Nikolić et al. 2026).

Furthermore, numerous observations of pondweeds are available in the Flora Croatica Database (FCD) (Nikolić et al. 2026). However, their utility for the present analysis proved limited for several reasons. Foremost, the taxonomic reliability of many records cannot be adequately verified because corresponding herbarium specimens are lacking. This is particularly problematic within the Potamogetonaceae, a taxonomically challenging group, characterised by frequent hybridisation and substantial morphological variability, often requiring detailed study for accurate identification (Kaplan et al. 2009, Kaplan 2010, Kaplan and Fehrer 2013, Aykurt et al. 2017, Fehrer et al. 2022). In addition, locality data associated with many records are imprecise or absent, especially where aquatic plants were documented incidentally during broader floristic surveys. Beyond the 12 pondweed taxa analysed in this study and the three previously discussed data-deficient species, two further taxa are known exclusively from literature sources lacking both herbarium vouchers and precise locality information: *P. filiformis* from Plitvice Lakes National Park (Krga 1992) and P. × fluitans from Papuk Nature Park (Pandža 2010). Owing to the absence of verifiable material and sufficiently detailed occurrence data, both records are considered doubtful. Although the authors of the present study conducted multiple research projects in Plitvice Lakes National Park over the years, *P. filiformis* has never been recorded there. Overall, this study constitutes the first comprehensive and systematic synthesis of the distribution of *Potamogeton* and *Stuckenia* in Croatia. Due to the methodological limitations of the WFD monitoring regarding the size and distribution of sampled waterbodies, further studies on macrophytes in Croatia would benefit from investigating smaller ponds, ditches, pools, intermittent streams and very large rivers.

## Conclusions

This study provides the first comprehensive and systematic overview of the distribution of *Potamogeton* and *Stuckenia* in Croatia, based on nine years of national surface water monitoring across a diverse range of lotic and lentic habitats. A total of twelve taxa were recorded, including ten *Potamogeton* species, one *Stuckenia* species and one *Potamogeton* hybrid, whose distribution patterns reflect the considerable environmental heterogeneity of Croatian waterbodies. The dominance of *P. nodosus* across all ecoregions and habitat categories underscores its exceptional ecological plasticity, which is further supported by its heterophyllous morphology and broad tolerance to physical and chemical water parameters. The frequent occurrence of *P. crispus* and *S. pectinata* highlights ongoing ecological pressures, particularly in the lowland watercourses of the Pannonian Ecoregion. Conversely, taxa such as *P. berchtoldii*, *P. natans* and *P. lucens*, occur more frequently in natural and Dinaric waterbodies. The rarity of certain taxa, including *P. coloratus*, P. gramineus and P. × cooperi, combined with the likely historical disappearance of boreal species, such as *P. alpinus* and *P. compressus*, reflects both the inherent biogeographic constraints of the region and the accelerating effects of climate change and habitat degradation. Importantly, the current WFD-based monitoring network, while robust for frequently occurring taxa, does not fully capture species associated with smaller, more isolated waterbodies, suggesting that targeted surveys of ponds, ditches and intermittent streams could substantially enrich our understanding of pondweed diversity in Croatia. The dataset compiled here constitutes a valuable baseline for long-term monitoring, macrophyte-based ecological status assessments and conservation planning of aquatic biodiversity in South-Eastern Europe.

## Supplementary Material

1062E9EF-FBEE-501D-809F-4A2397A1DC7A 10.3897/BDJ.14.e208115.suppl1 Supplementary material 1 STable S1 Data type site information Brief description Descriptive statistic of watercourse sampling sites with pondweeds occurring in more than two localities. The percentage indicates the proportion of the total number of surveyed sites in which a given taxon was recorded (NM – *Nuphar–Myriophyllum* type, BN – *Berula–Nasturtium* type, My – *Myriophyllum* type, Po – *Potamogeton* type, HC – *Hydrocharis–Ceratophyllum* type, RF – *Rhynchostegium–Fontinalis* type; P - Pannonian Ecoregion, CD - Continental - Dinaric Subecoregion, MD - Mediterranean-Dinaric Subecoregion; x - significant fluctuations in water flow; PotNod – *Potamogeton nodosus*, PotCri – *Potamogeton crispus*, StuPec – *Stuckenia pectinata*, PotPer – *Potamogeton perfoliatus*, PotBer – *Potamogeton berchtoldii*, PotLuc – *Potamogeton lucens*, PotNat – *Potamogeton natans*, PotPus – *Potamogeton pusillus*, PotTri – *Potamogeton trichoides*). File: oo_1717014.xlsx https://binary.pensoft.net/file/1717014 Marija Bučar

78FA3A84-FB6C-5C01-920A-F4A1F86B67DC 10.3897/BDJ.14.e208115.suppl2 Supplementary material 2 SuppTable S2 Data type site information Brief description Information on analysed watercourse sampling sites with pondweeds (NM – *Nuphar–Myriophyllum* type, BN – *Berula–Nasturtium* type, My – *Myriophyllum* type, Po – *Potamogeton* type, HC – *Hydrocharis–Ceratophyllum* type, RF – *Rhynchostegium–Fontinalis* type; P – Pannonian Ecoregion, CD – Continental-Dinaric Subecoregion, MD – Mediterranean-Dinaric Subecoregion; x – significant fluctuations in water flow). File: oo_1717020.xlsx https://binary.pensoft.net/file/1717020 Marija Bučar

6823749B-27DE-53D5-8C30-8566C3FA07A7 10.3897/BDJ.14.e208115.suppl3 Supplementary material 3 Table S3 Data type site information Brief description Information on analysed standing waterbodies sampling sites where pondweeds were recorded (P – Pannonian Ecoregion, CD – Continental-Dinaric Subecoregion, MD – Mediterranean-Dinaric Subecoregion; PotNod – *Potamogeton nodosus*, PotCri – *Potamogeton crispus*, StuPec – *Stuckenia pectinata*, PotPer – *Potamogeton perfoliatus*, PotBer – *Potamogeton berchtoldii*, PotLuc – *Potamogeton lucens*, PotNat – *Potamogeton natans*, PotPus – *Potamogeton pusillus*, PotTri – *Potamogeton trichoides*). File: oo_1717017.xlsx https://binary.pensoft.net/file/1717017 Marija Bučar

E8354388-EC20-528D-9A12-B72D9AFFC190 10.3897/BDJ.14.e208115.suppl4 Supplementary material 4 Table S4 Data type occurrences Brief description A machine-readable file containing occurrences of Potamogetonaceae taxa in studied sites. File: oo_1754477.csv https://binary.pensoft.net/file/1754477 Marija Bučar

## Figures and Tables

**Table 1.** Distribution of standing water sampling sites with recorded pondweeds across environmental categories. The percentage indicates the proportion of the total number of natural or significantly modified and artificial lakes. (P – Pannonian Ecoregion, CD – Continental–Dinaric Subecoregion, MD – Mediterranean–Dinaric Subecoregion).

		Number of natural lakes (6)	Number of significantly modified and artificial lakes (26)
Area (km^2^)	< 1	3 (50.0%)	10 (38.5%)
	1 - 10	2 (33.3%)	16 (61.5%)
	10 - 100	1 (16.7%)	-
Average depth (m)	< 3	1 (16.7%)	10 (38.5%)
	3 - 10	-	16 (61.5%)
	10 - 15	2 (33.3%)	-
	> 15	3 (50.0%)	-
Altitude (m a.s.l.)	< 200	4 (66.7%)	18 (69.2%)
	200 - 500	-	5 (19.2%)
	> 500	2 (33.3%)	3 (11.5%)
(Sub)Ecoregion	P	-	11 (42.3%)
	CD	2 (33.3%)	2 (7.7%)
	MD	4 (66.7%)	13 (50.0%)
